# Chromatographic immunoassays for Helicobacter pylori detection – are they reliable in Mali, West Africa?

**DOI:** 10.11604/pamj.2013.14.72.2131

**Published:** 2013-02-20

**Authors:** Ingvild Austarheim, Kari Tvete Inngjerdingen, Berit Smestad Paulsen, Adiaratou Togola, Chiaka Diakité, Drissa Diallo

**Affiliations:** 1 School of Pharmacy, University of Oslo, P.O. Box 1068 Blindern, 0316 Oslo, Norway; 2 Department of Traditional Medicine, BP 1746, Bamako, Mali

**Keywords:** Stool test, serological test, rapid tests, Helicobacter pylori

## Abstract

**Introduction:**

Gastrointestinal diseases are major reasons for morbidity in Mali. As *Helicobacter pylori* is known to play a major role in gastritis and gastric ulcer we wanted to find a simple method for detection.

**Methods:**

Twenty-nine volunteers with confirmed gastric ulcer by gastroscopy and 59 randomly selected volunteers were diagnosed by using the rapid serological test Clearview^®^ *H. Pylori*. The ImmunoCard STAT!^®^ HpSA^®^test was applied on stool from 65 volunteers seeking help for gastrointestinal related ailments.

**Results:**

A *Helicobacter pylori* prevalence of 21% was found among the individuals with confirmed gastric ulcer, 44% among the randomly selected volunteers and 14% in individuals with gastrointestinal related ailments.

**Conclusion:**

According to what is already known about the aetiology of gastric ailments and the prevalence of *Helicobacter pylori* in neighboring countries, the infection rates in our study appear strikingly low. This might indicate that Clearview^®^ *H. Pylori* and ImmunoCard STAT!^®^ HpSA^®^ have low sensitivities in the populations studied. Strain variability of *H. Pylori* may be an explanation. The tests need to be properly evaluated in Mali before they can be relied upon as diagnostic tools.

## Introduction

Gastritis and other gastrointestinal (GI) disorders are important causes of morbidity in the African population [1]. In Mali, the prevalence of gastric ulcer in the population is reported to be 4.2% for men and 2.4% for women, and is probably higher for gastritis and non-ulcer dyspepsia [2, 3]. Medicinal plants are popular remedies for treating gastrointestinal ailments in Mali, as approximately 75% of the population mainly use plant based medicines for their primary health care [2, 4, 5]. It is important to increase the knowledge behind the aetiology of gastritis, gastric ulcer and GI-related problems in Mali for future research on Malian traditional medicines used for these ailments, and also in order to give patients the correct treatment.The presence of *Helicobacter pylori* in the population is one important factor needing assessment, as *H. Pylori* is a known factor contributing to dysfunction of the GI.

Individuals may be asymptomatic carriers of the disease, but the presence of *H. Pylori* is highly correlated with underlying ailments causing dyspepsia. Infections with *H. Pylori* are found worldwide in individuals of all ages, but are commonly acquired at an earlier age in developing countries [6]. A previous study conducted in Mali on patients with gastric ulcer reported an *H. Pylori* prevalence of 95% [7]. The prevalence of *H. Pylori* in dyspeptic patients in other West African countries, diagnosed by biopsy and urease test, is reported to be 97% in Ghana [8], 91.3% in Ivory Coast [9], and 72-91% in Nigeria [10, 11]. In Senegal a prevalence of 100% was found in patients with ulcers or gastritis by urea breath test and histological findings [12].

Rapid stool- or serology-based chromatographic immunoassays were chosen in this study, as we wanted to diagnose patients in Mali with a simple, non-invasive technique.

## Methods

### Volunteers with confirmed gastric ulcer

Twenty-nine volunteers diagnosed with gastric ulcer, 18 (62%) male and 11 (38%) female, confirmed by gastroscopy at Cabinet Medical “Les étoiles” at Gabriel Touré hospital, Bamako, were tested by the chromatographic immune based assay Clearview^®^ *H. Pylori* (Alere Inc., Waltham, MA). Nine different ethnic groups were included; Bambara (31%), Peulh (28%), Sénoufo, Malinké, Somono, Dogon, Dafing, Sonrhaï and Minianka. The average age was 41 years (age 27 - 65 years). *H. Pylori* antibodies (IgG) present in fresh whole blood were analysed according to the manufacturer's instructions. Briefly, a thin lancet was used to puncture the skin of the finger and 50 µl whole blood and one drop of buffer was dispensed into the round window of the test device. The assay was carried out at room temperature (30°C), and was read after 10 minutes. A pink-red line, even very weak, was considered as a positive result. All volunteers gave informed consent, confirmed by a signature.

### Randomly selected volunteers

The chromatographic immune based assay Clearview^®^H. pylori was used to detect *H. pylori* antibodies (IgG) present in fresh whole blood of 59 volunteers. The volunteers were randomly recruited from three different areas of Bamako, Mali; Hippodrome, Daoudabougou and Sotuba. The group included 31 (53%) female and 28 (47%) male with an average age of 36 years (age 18 - 65 years). Fourteen different ethnic groups were involved; Bambara (24%), Malinké (14%), Bozo, Dafing, Haoussa, Kakolo, Peulh, Sarakolé, Sénoufo, Sonrhaï, Tamasheq, Fofana, Sangare and Minianka. Whole blood was analysed as described under subsection 2.1. All volunteers gave informed consent, confirmed by a signature or a fingerprint.

### Volunteers with diverse gastric ailments

The chromatographic immune based assay ImmunoCard STAT!^®^HpSA^®^ Stool antigen test (Meridian Bioscience Inc., Cincinnati, OH) was used to detect H. pylori antigens present in the stool of 65 volunteers. The stool samples were given at the INRSP (Institut National de Recherche en Santé Publique, Bamako) due to ailments like infections (33%), dyspepsia (16%) and other stomach related ailments. The group included 33 (52%) female and 31 (48%) male, and with an average age of 33 years (age 2 - 70 years). Thirteen different ethnic groups were included; Sarakolé (23%), Peulh (20%), Bambara (19%), Sénoufo, Dogon, Malinké, Kasonké, Kakolo, Maure, Minianka, Diawando, Sonhraï and Somono. All stool samples had a pasty consistency and they were analysed at room temperature (25°C) two hours after stool sampling, according to the manufacturer's guidelines. Briefly, four drops of diluted stool were dispensed into the round window of the test device and the result was read after five minutes of incubation at room temperature. The appearance of a pink-red band in the reading window, even very weak, was considered as a positive result. All volunteers gave an informed consent for bacteriological research on stool samples.

### Ethical approval

This work was a research project integrated in a diagnostic process at INRSP and in research program on traditional anti-gastric ulcer medicinal plants. For this reason, the project on its own was not presented to the ethic Committee of INRSP. Prior consent was obtained from all participants before taking or using samples.

## Results

### Volunteers with confirmed gastric ulcer

*H. pylori* antibodies were detected in 21% of the 29 volunteers diagnosed with gastric ulcer by gastroscopy (Table 1). These individuals were tested directly after endoscopy, and had not been through *H. Pylori* eradicating regime. No association with a positive detection of *H. Pylori* and gender, age or ethnic group was found (**Additional material A**).

**Table 1:** Results obtained from serological and stool based tests in *Helicobacter pylori* detection

	Positive (%)	95% CI	n
Confirmed gastric ulcer (serology based)	21	(6;35)	29
Randomly selected volunteers (serology based)	44	(31;57)	59
Gastric ailments (stool based)	14	(5;22)	65

95% CI; 95% Confidence interval, n; No of volunteers

### Randomly selected volunteers

*H. pylori* antibodies were detected in 44% of the randomly selected volunteers (Table 1). No association between *H. Pylori* positive individuals, gender, age or ethnic group was found. Nine (15.2%) individuals reported that they had previously undergone gastroscopy; out of these two were *H. Pylori* positive in the present study. Thirty-six (61%) of the volunteers reported that they from time to time experienced dyspepsia, mostly due to Gastroesophageal Reflux Disease (GARD), and of these individuals 36% were *H. pylori* positive. Among the volunteers who did not experience GI symptoms, 54% were *H. pylori* positive. Four of the volunteers knew that they had recently been taking antibiotics, and of these three were *H. Pylori* positive. See **Additional material B** for additional data on gender, age, ethnicity and GI related problems.

### Volunteers with diverse gastric ailments

*H. Pylori* antigens were present in 14% of the volunteers suffering from diverse gastric ailments (Table 1). Of the nine individuals that were *H. Pylori* positive, five were tested for gastrointestinal infections problems. None of the dyspeptic individuals tested positive (**Additional material C**).

## Discussion

According to the manufacturer, Clearview^®^ *H. pylori* is regarded as a sensitive test (2), and a negative result should therefore indicate that the person truly is negative. In the present study we found that only 21% of the individuals with confirmed gastric ulcer tested positive for *H. pylori* by using Clearview^®^ *H. pylori*. This number is probably too low to be realistic as it does not correlate with a previous investigation carried out in Mali in year 2000. Biopsies taken from 121 patients diagnosed with gastric ulcer by endoscopy confirmed that *H. Pylori* were present in 95% of the cases [7]. The population studied came from the same area (Bamako) and the gastric ulcer was diagnosed by endoscopy in the same manner as in the present study. The culture and habits have not changed much in Mali the last decade, which means that the populations studied should be comparable. It would therefore be expected that most of the individuals diagnosed with gastric ulcer in the current study should be *H. pylori* positive.

A low (41%) prevalence was also shown by Clearview^®^ *H. pylori* among the randomly selected volunteers. The *H. pylori* sero-prevalence of healthy individuals in nearby countries is reported to be much higher, 80 - 85% in Nigeria [13] and 75.4% in Benin [14]. In addition, random studies in Africa have shown a seroprevalence of 80% [1]. Factors that seem to play a role in the infection rate are low-quality drinking water, lack of basic hygiene, habit of eating from the same plate as well as poor diet and overcrowding [11, 15]. These factors are similar between Mali and the surrounding countries, making a comparable prevalence highly likely. To our comprehension, the most obvious reason to explain the low detection of *H. Pylori* is that Clearview^®^ *H. pylori* has a low sensitivity in the population studied in Mali. Sensitivity is not an intrinsic property of an assay [16], and it is therefore possible that the sensitivity experienced in the population studied here, is not the same as for the population studied by the manufacturer.

A low prevalence of *H. pylori* (14%) was also found by the ImmunoCard STAT!^®^HpSA^®^ stool test. A good practice in diagnosing *H. pylori* with antigen tests is that antibiotics should be stopped at least four weeks and anti-secretory therapy at least two weeks before testing, as these medications negatively affects the sensitivity [11]. In Mali malaria is endemic, and anti-malaria drugs might have a negative effect on the sensitivity of the test, as these drugs might eradicate or decrease the presence of *H. pylori* [17, 18]. Most of the individuals in our study have a low educational level, and it was therefore difficult to know if they had been taking any interfering medications. *H. pylori* is, however, not easily eradicated [19], and self-medication is probably not sufficient for total removal of the bacteria, but potentially enough to lower the amount of stool antigens to make the outcome negative.

According to Meridian Bioscience, it is a possibility that the stool test does not respond to all subgroups of *H. Pylori*. There is a remarkable genetic diversity of *H. Pylori* worldwide, the number of genetic variability of *H. Pylori* exceeding 2 to 5-fold the variability of other bacterial pathogens. Substantial diversity has recently also been described for the important virulence factor cagA [20]. This means that ImmunoCard STAT!^®^HpSA^®^, which uses monoclonal antibodies for detection, might have a lower sensitivity towards antigens produced by some strains. Calvet et al. [21] reported a sensitivity of 69-74% for ImmunoCard STAT!^®^HpSA^®^ on patients with dyspeptic symptoms. This is much lower then what reported by the manufacturer (Table 2).

**Table 2:** Test sensitivities of the utilized serological and stool based tests as given by the manufacturer

	Test sensitivity	95% CI
Clearview^®^ (serology based)	93.0	(87.1;96.7)
ImmunoCard STAT!^®^ HpSA^®^ (stool based)	96.1	(86.5;99.5)

95% CI; 95% Confidence interval

A weakness in the present study might be that the serological and the stool tests were not carried out on the same individuals, thereby making it difficult to compare these methods directly. However, our results suggest that both assays have a considerably lower sensitivity in Mali than reported by the manufacturers. A follow-up study is needed in order to give any definite conclusions. It is noteworthy that the majority (61%) of the randomly selected volunteers experienced gastrointestinal symptoms. This reflects the high degree of GI related ailments experienced by the population of Mali. Natural products which reduce the density of *H. pylori* colonization and mucus adherence [22], and thereby lower the dyspeptic symptoms, might be highly relevant for further research as rapid recurrence and antibiotic resistance are increasing problems in countries with high a prevalence of *H. pylori* [23]. In addition, as Mali is ranked as one of the poorest countries in the world, the population has medicinal plants as the main option for most of primary health care. It is therefore highly important to carry out more research on traditional medicines used for these types of ailments, and also to have access to simple and non-expensive diagnostically methods reliable for the Malian population.

## Conclusion

Rapid immunoassays for *H. Pylori* detection need to be properly evaluated in the respective countries where they are to be used before they can be relied upon as diagnostic tools. Interfering circumstances such as medications and strain variability should be addressed in greater detail. Due to endemic malaria, strain variability and self medication, it is a possibility that the stool based test loses some of its sensitivity. As self medication is difficult to avoid, it is probably wise to choose a different test system for *H. Pylori* identification in Mali.

## Additional material

**Additional material A: - Pdf(21 Kb)**: Confirmed gastric ulcer by gastroscopy (serological test, Clearview^®^ *H. pylori*)

**Additional material B - Pdf(21 Kb)**: Randomly selected volunteers (serological test, Clearview^®^ *H. pylori*)

**Additional material C - Pdf(21 Kb)**: Volunteers seeking help for diverse gastric ailments (stool test, ImmunoCard STAT!^®^HpSA^®^)

http://www.panafrican-med-journal.com/content/article/14/72/full/annex/annex.pdf
